# Annual acknowledgement of manuscript reviewers

**DOI:** 10.1186/1749-8546-8-6

**Published:** 2013-03-20

**Authors:** Siu-wai Leung

**Affiliations:** 1 International Society for Chinese Medicine, Macao, China

## Abstract

**Contributing reviewers:**

The editors of *Chinese Medicine* would like to thank all our reviewers who have contributed to the journal in Volume 7 (2012).

## 

Hyai Khan Abdul

United States of America

Denise Adams

Canada

Adeolu Adedapo

Nigeria

Luis Afonso

Brazil

Lei An

China

Reine Raïssa Aworet Samseny

Gabon

Zhaoxiang Bian

Hong Kong

Christopher Branford-White

United Kingdom

Adam Burke

United States of America

Raymond Chuen-Chung Chang

Hong Kong

Jin Chen

China

Xiuping Chen

China

Fang-Pey Chen

Taiwan

Din Chen

United States of America

Chung-wah Cheng

Hong Kong

Jen-Hwey Chiu

Taiwan

William CS Cho

Hong Kong

I-min Chu

Taiwan

Cuneyt Cirak

Turkey

Thiago Cunha

Brazil

Maoxian Deng

China

Hongmei Duan

China

Heloina Falcão

Brazil

Yutong Fei

China

Yi Feng

China

Yibin Feng

Hong Kong

Arthur Sa Ferreira

Brazil

Johannes Fleckenstein

Germany

Sybille Franke

Germany

Qinmin Ge

China

Qiang Ge

United States of America

Fatemeh Ghaffarifar

Iran

William Goggins

Hong Kong

Ram Gordon

United States of America

Guangyi Xiong

China

Fernando Guimaraes

Brazil

Jingchun Guo

China

Deliang Guo

United States of America

Madan Gupta

India

Sabine Guth

Germany

Gloria Gutiérrez-Venegas

Mexico

Michio Hashimoto

Japan

Chengwei He

Macao

Bonilla-Jaime Herlinda

Mexico

Hongtao Jin

China

Heng Li Huang

Taiwan

Weng-foung Huang

Taiwan

Jung-Hee Jang

South Korea

Cizhong Jiang

China

Zhi-Hong Jiang

Macao

Lin Jiang

United States of America

Takenobu Katagiri

Japan

Syed Khundmiri

United States of America

Dae OK Kim

South Korea

Jochen Klein

Germany

Alex KleinJan

Netherlands

Jun Kong

China

Henry Lam

Hong Kong

Jean-Marc Lavoie

Canada

Myeong Soo Lee

South Korea

Simon Ming Yuen Lee

Macao

Tzung-Yan Lee

Taiwan

Siu Man Leung

Hong Kong

Ping-Chung Leung

Hong Kong

Kelvin Leung

Hong Kong

Siu-wai Leung

Macao

Shao Li

China

Peng Li

Macao

Jiang Lin

China

Bi-Fong Lin

Taiwan

Zhao Ling

China

Chenghai Liu

China

Airong Liu

China

Jia-Jun Liu

China

Aiping Lu

China

Guanghua Lu

China

Jingyuan Mao

China

Sergio Martinez-Luis

Panama

Pulok Mukherjee

Iraq

Johannes Novak

Austria

Byeongsang Oh

Australia

Milton Oliveira

Brazil

KT Oo

United States of America

Laurence Ozmen

Switzerland

Chunliu Pan

United States of America

Deepti Pandita

India

Gerald Pang

Australia

Prahlad Parajuli

United States of America

Wen-Huang Peng

Taiwan

Can Peng

United States of America

Jiri Petrak

Czech Republic

Souwalak Phongpaichit

Thailand

Hailin Qin

China

Srikanta Rath

India

Anastasia Rowland-Seymour

United States of America

Helton Santiago

United States of America

Elisabetta Schievano

Italy

Pang Chui Shaw

Hong Kong

Chi Hyun Song

South Korea

Pablo Steinberg

Germany

Sanan Subhadhirasakul

Thailand

Mohd Roslan Sulaiman

Malaysia

Anna Szakiel

Poland

Shusheng Tai

Canada

Nozomi Takahashi

Belgium

Kenneth To

Hong Kong

Selma Tobudic

Austria

Karl WK Tsim

China

Jaleh Varshosaz

Iran

Loganathan Veerappan

Malaysia

Jian-Bo Wan

Macao

Jianguo Wang

Australia

Yi Wang

China

Yan wang

China

Sheng Wei

China

Zhaojun Wei

China

Ricky Wong

Hong Kong

Taixiang Wu

China

Chunfu Wu

China

Jian-lin Wu

Macao

Feiyue Xing

China

Fengguo Xu

Singapore

Shikai Yan

China

Hin Wing Yeung

Macao

Jia Yongliang

China

Jian You

China

Yusmazura Zakaria

Malaysia

Anthony Zhang

Australia

Shouqin Zhang

China

Nevin L Zhang

Hong Kong

Meixia Zhang

China

Grant Zhang

United States of America

Ying Zheng

Macao

Xiaobo Zhou

United States of America

Qing Zhou

United States of America

